# Brainstem Glioma in Adults

**DOI:** 10.3389/fonc.2016.00180

**Published:** 2016-08-09

**Authors:** Jethro Hu, Stephen Western, Santosh Kesari

**Affiliations:** ^1^Department of Neurology, Cedars-Sinai Medical Center, Los Angeles, CA, USA; ^2^Department of Neurosurgery, Cedars-Sinai Medical Center, Los Angeles, CA, USA; ^3^Independent Researcher, Vancouver, Canada; ^4^Department of Translational Neuro-Oncology and Neurotherapeutics, John Wayne Cancer Institute, Pacific Neuroscience Institute, Providence Saint John’s Health Center, Santa Monica, CA, USA

**Keywords:** DIPG, glioma, IDH mutations

## Abstract

Brainstem gliomas are not nearly as common in adults as they are in children. They are likely the final common consequence not of a single disease process but of several. They can be difficult to diagnose, and are challenging to treat. Clinical studies of this diagnosis are few and generally small. Because of these factors, our understanding of the biology of adult brainstem glioma is incomplete. However, the knowledge base is growing and progress is being made. In this article, we review the current state of knowledge for brainstem glioma in adults and identify key areas for which additional information is required.

## Introduction

The past several years have yielded important insights into the biology of glioma in adults. Efforts such as The Cancer Genome Atlas (TCGA) have comprehensively cataloged the litany of somatic alterations occurring in glioblastoma and lower grade gliomas, leading to discoveries such as the importance of *IDH1* mutations in the development of a low-grade glioma and secondary glioblastoma (1). Pediatric diffuse intrinsic pontine glioma (DIPG) – the most frequent malignant primary brain tumor of childhood – on the other hand, has been found to have substantially different biological underpinnings. Eighty percent of these tumors, for example, contain mutations in genes that encode histone H3 (2).

At the nexus of these two diagnoses is brainstem glioma in adults. Unfortunately, it is perhaps more apt to describe adult brainstem glioma as the valley shrouded in shadow between the two growing mountains of knowledge that represent adult glioma and pediatric DIPG, as few studies have investigated this particular diagnosis. The anatomical basis of adult brainstem glioma does not lend itself to easy study, as the brainstem is packed full of brainstem nuclei and white matter tracts that are essential for basic functions. Resection is often not possible; even biopsies are challenging with significant risk for complications.

Within the umbrella of adult brainstem glioma, there is a subset of tumors that is similar in appearance and potentially similar in etiology to childhood DIPG, but with slightly older onset, generally affecting young adults. There is another subset of more well-circumscribed lesions that shares clinical and radiographic features with pilocytic astrocytoma, also with a younger median age at onset. The subset of adult brainstem gliomas presenting in later adulthood are typically more akin to other *de novo* malignant gliomas of adulthood. Yet despite these differences, there are also commonalities between these entities in terms of clinical considerations.

In this article, we will review the current state of knowledge (and lack of knowledge) for adult brainstem glioma and highlight prospects for advances in therapy.

## Clinical Features

### Appearance and Presentation

In contrast to pediatric DIPG, which accounts for approximately 20% of pediatric primary brain neoplasms, adult brainstem glioma constitutes less than 2% of adult gliomas, with a slight male preponderance (3, 4). Median age at diagnosis is in the mid-30s, though brainstem gliomas can present in any decade of life.

As befits a diagnosis that encompasses an array of pathophysiologic alterations, the radiographic appearance of adult brainstem glioma varies widely, with approximately 40% demonstrating enhancement (3, 5). By comparison, in pediatric DIPG, contrast enhancement is usually not a prominent feature. Contrast enhancement itself can take on a variable appearance, with some tumors exhibiting minimal or partial enhancement, and others showing robust enhancement. MR spectroscopy can be a useful aid for diagnosis, as elevation of the choline/NAA ratio is often detectable, with one analysis of adult brainstem gliomas showing this finding in 100% of cases analyzed (3). FDG–PET may also be helpful in differentiating aggressive lesions from indolent ones (6).

**Figure 1:**
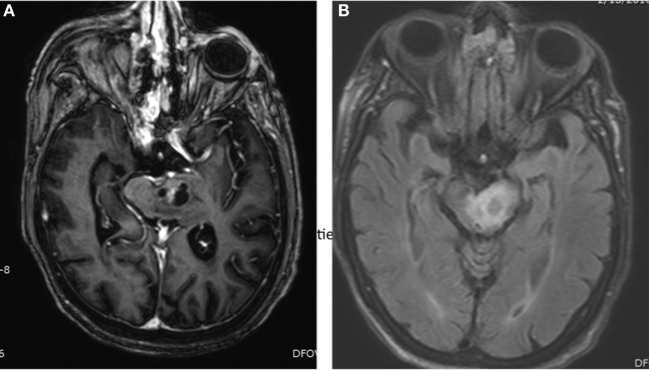
**Brain MRI showing presence of brainstem glioma in a 82-year-old-woman with histological diagnosed astrocytoma, grade III**. **(A)** Axial, post-gadolinium MRI sequences showing a cystic, enhancing left pontine mass. **(B)** Axial, FLAIR MRI sequences showing surrounding edema in the left pontine mass.

Brainstem gliomas are centered in the pons in approximately 60% of cases, but can also arise from the midbrain or medulla, and can infiltrate beyond the brainstem (7). They can be exophytic or expansile on imaging, or, as is the case with classic DIPG, can be infiltrative and diffuse with little notable mass effect (Figure 1).

Clinically, it is important to distinguish between gliomas that involve the midbrain tectum – which often behave indolently – and classic diffuse pontine gliomas, which often do not enhance with contrast or have varying enhancement patterns. Tectal gliomas are typically low-grade, non-enhancing, and often non-progressive. They are diagnosed most frequently in childhood, but can be detected at any age. Observation is often a reasonable management option, and when intervention is warranted, often it is only to address obstructive hydrocephalus. A small subset of tectal glioma may behave more aggressively; contrast enhancement and the development of cystic changes may herald such progression (8). Because of their distinct natural history, tectal gliomas are generally not considered in the same vein as other brainstem gliomas, and will not be further addressed in this review.

Histologically, adult brainstem gliomas can have an astrocytic, oligodendroglial, or mixed appearance, with astrocytic tumors further characterized as either pilocytic or diffusely infiltrative. A single-institution Italian retrospective analysis of 21 patients with histologically confirmed disease identified 2 pilocytic astrocytomas, 9 low-grade astrocytomas, 8 anaplastic astrocytomas, and 1 glioblastoma (3). An MD Anderson retrospective analysis that included 98 cases with histology identified 28 glioblastomas, 43 anaplastic astrocytomas, 15 diffuse astrocytomas, and 11 gliomas not otherwise specified (5).

The most frequent presenting symptom for adult brainstem glioma is headache, which can be a manifestation of hydrocephalus. Cranial nerve deficits and long tract signs are also common. “Crossed” deficits, in which facial signs and symptoms are contralateral from arm/leg signs and symptom, are another characteristic hallmark of brainstem pathology. Occasionally, clinical decline can precede radiographic progression, as the density of critical structures in the brainstem leaves little margin for growth before deficits occur.

### Prognostic Considerations

Whereas pediatric DIPG is associated with a dismal prognosis of 10 months, with only 10% of pediatric patients living >2 years beyond diagnosis, median survival for adult brainstem glioma is in the grim-but-not-quite-as-dismal range of 30–40 months (5). Furthermore, because adult brainstem gliomas vary widely in their aggressiveness, prognosticating outcome for an individual patient at diagnosis can be difficult.

Increasing tumor grade and contrast enhancement are associated with significantly reduced survival. An analysis of 17 adults with DIPG demonstrated a median OS of 57 months for low-grade vs. 16 months for high-grade gliomas (9). Contrast enhancement correlated strongly with histological grade in this study – median OS in patients with non-enhancing DIPG was 57 vs. 13 months for patients with enhancing tumors. The MD Anderson retrospective analysis of adult brainstem glioma patients demonstrated a median OS of 77.0 months for WHO grade 2 diffuse astrocytoma, 21.1 months for WHO grade 3 anaplastic astrocytoma, and 14.8 months for glioblastoma (5).

Increasing age is also associated with worsened survival. The MD Anderson retrospective study demonstrated median survival of 34 months for patients 22–59 years of age, but only 14.2 months for patients 60 and over (5).

Other characteristics that have been identified as favorable prognostic factors include duration of symptoms >3 months, and the presence of “necrosis” on MRI (9, 10). Location also factors into prognosis – the MD Anderson study demonstrated a median survival of 66.0 months for midbrain tumors, 25.3 months for pontine tumors, and 51.3 months for medullary tumors (5). Predisposing factors are not well-defined, though there is one case report of a brainstem glioma developing 8 years after radiation treatment for a pituitary adenoma (11).

### Treatment Options

For pediatric DIPG, standard of care treatment consists of involved field radiation therapy, typically to a dose of approximately 54–60 Gy. Alternative fractionation schemes for pediatric DIPG – including hyperfractionated, accelerated, and hypofractionated regimens – do not improve survival, with significantly increased risks of radiation toxicity at doses >64 Gy (12).

To date, pediatric treatment trials for DIPG have failed to identify a significant benefit with chemotherapy. A phase 2 trial of the oral alkylating drug temozolomide along with radiation followed by adjuvant temozolomide – similar to the standard Stupp regimen for adult malignant glioma – resulted in median OS of only 9.5 months (13).

Other failed strategies in the pediatric DIPG population include high-dose chemotherapy, and metronomic approaches with etoposide, trophosphamide, or temozolomide (14–16). Several phase I and phase II trials of targeted therapy have been conducted, including trials of anti-EGFR drugs such as erlotinib, gefitinib, and nimotuzumab; trials targeting PDGFR using imatinib; trials utilizing the farnesyltransferase inhibitors tipifarnib and lonafarnib; trials of antiangiogenic drugs such as bevacizumab, semaxanib and the integrin inhibitor cilengitide; a trial of the mTOR inhibitor everolimus; and a trial of the multikinase inhibitor vandetanib (17–27). Overall, the results of these trials have been unimpressive, though long-term disease control has been reported in a small subset of these patients.

For adults with brainstem glioma, involved field radiation therapy, typically to a dose of 54–60 Gy, is considered standard upfront therapy, as is the case with pediatric DIPG. Few studies utilizing chemotherapy for the treatment of adult brainstem glioma have been performed, and the ones that have are often limited to single-arm, single-institution, or retrospective analyses. However, there is reason to hope that chemotherapeutic treatment for adults is more fruitful. The final results of RTOG 9802 demonstrate a clear benefit for combined chemoradiation vs. radiation therapy alone for patients with so-called “high-risk” grade 2 glioma, defined in this study as patients older than 40 or who had undergone subtotal resection. The benefits of combined treatment were particularly striking in patients with tumors that harbor an IDH1 mutation. Although the recently published results do not delineate how many of the 251 patients enrolled on this study had brainstem disease, the study adds to the body of evidence supporting a combined treatment approach for adult glioma patients of any age.

Perhaps the best line of evidence supporting the use of upfront chemotherapy (along with radiation) for adult brainstem glioma comes from the MD Anderson retrospective analysis, which demonstrated a striking difference in survival in adults with brainstem glioblastoma treated with the standard Stupp regimen (concurrent radiation and temozolomide followed by adjuvant temozolomide) vs. those who were not – median OS for patients treated with the Stupp regimen was 23.1 vs. 4.0 months for patients who were not (5). Of course, the scope of this analysis is limited by its small numbers (28 patients) and retrospective nature, as patients treated with the Stupp regimen tended to have better performance status at baseline (KPS 90 vs. 80), and were treated in the temozolomide era in which other advances in treatment and management may influence survival. A single-institution retrospective analysis of 15 adults with “low grade” diffuse brainstem glioma (defined either histologically or by radiographic appearance) treated with temozolomide at recurrence demonstrated a median PFS of 9.5 months and median OS of 14.4 months (9). Clinical improvement was noted in 60% of patients, and radiographic responses occurred in 6 of 15 patients (4 partial and 2 minor responses).

Re-irradiation is another potential treatment strategy for recurrent adult brainstem gliomas. A single-institution retrospective analysis of five adults with progressive/recurrent brainstem glioma treated with a repeat course of radiation resulted in post-treatment survival ranging from 3 to 36+ months (28). Four of the five patients showed improvement in performance status post-treatment, with the other patient manifesting new symptoms potentially attributable to radiation toxicity.

The antiangiogenic drug bevacizumab may also have a role in the management of adults with brainstem glioma. A retrospective analysis that included 17 patients treated with bevacizumab at progression demonstrated median PFS of 2.0 months with 6-month PFS of 21%, including one patient who was progression free for 9 months (5). While somewhat encouraging, the 6-month PFS of 21% pales in comparison to the 40% 6-month PFS seen with supratentorial glioblastoma. It is also worth noting that bevacizumab may have a palliative benefit in addition to its anti-neoplastic activity. Bevacizumab reduces vasogenic cerebral edema, which frequently results in symptomatic improvement. Furthermore, this effect often allows patients to taper their steroid dose, which can have downstream benefits as well. Because bevacizumab both vasoconstricts and reduces vascular permeability, treatment with bevacizumab often mitigates contrast enhancement. Monitoring for changes on T2/FLAIR and Diffusion-Weight Imaging (DWI) takes on additional importance in this circumstance.

## Scientific Understanding

Few scientific studies have specifically investigated brainstem glioma in adults, but our understanding of the biology of pediatric DIPG has been greatly enhanced over the past few years by several landmark studies (Table 1). In one such study published in 2012, Wu et al. identified mutations in *H3F3A* and *HIST1H3B* in 78% of pediatric DIPG (2). The mutations in both of these genes result in a K27M amino acid substitution in histone H3, and suggest epigenetic dysregulation as an important contributor to the pathogenesis of pediatric DIPG. In contrast, histone H3 mutations rarely occur in adult supratentorial glioblastoma (29).

**Table 1:** **Summary of adult and pediatric brainstem gliomas**.

	Pediatric DIPG	Adult brainstem glioma	Adult supratentorial GBM
Incidence	20% of pediatric brain tumors	2% of adult glioma	80% of primary malignant brain tumors
Median age of onset	5–9	mid-30s	64
MRI contrast enhancement	Usually nonenhancing	Variable enhancement	Strong enhancement
Significant gene alterations	HIST1H3B	IDH1	MGMT promoter methylation
	H3F3A (K27M)	H3F3A (K27M)	EGFR amplification
	TP53		IDH1 and IDH2
	AVCR1		TERT promoter
	PPM1D		
	MYC		
	NTRK fusions		
Standard initial treatment	RT	RT	RT and temozolomide
Median overall survival	10 months	30–40 months (varies by grade)	12–18 months

Somatic *IDH* mutations, on the other hand, are very common in adult non-brainstem lower-grade (grade 2/3) gliomas. In the TCGA analysis of such tumors, 80.1% were found to harbor *IDH* mutations. These mutations were previously thought to be uncommon in adult brainstem glioma, as studies of pediatric DIPG did not identify any such mutations, and *IDH1 R132H* mutations as detected by immunohistochemistry were found in only 6% of the adult brainstem gliomas in one study (5). However, a whole-exome sequencing study of brainstem glioma demonstrated that *IDH* mutations are not altogether uncommon in the subset of tumors from adults, particularly when alternative non-*R132H* mutations (which constitute only 5% of *IDH* mutations in adult supratentorial glioblastoma) are included (30). Of the 13 adult brainstem gliomas that underwent whole exome sequencing in this study, 5 (38%) were *IDH1* mutated. There were three *IDH1 R132H* mutations, one *R132C*, and one *R132L* mutation. All of these cases also had mutant *TP53*, and four of the cases had mutant *ATRX*. Genetically, this group resembles classic *IDH1* mutant cerebral astrocytomas, which also usually harbor mutations in *TP53* and *ATRX*. Mean age of these 5 patients was 40 and the predominant tumor location was in the pons, with all 5 having a pontine location, four of which were located both in the pons and medulla. Histopathology was oligoastrocytoma grade 2 or 3 in four cases and GBM in one. *IDH1*-mutant cases had a significantly better median survival than the *IDH1* wild-type cases.

In the same study, *H3F3A* mutations were identified in 7/13 (54%) tumors. Each of these seven cases additionally harbored a mutation in either *TP53* (four cases) or *PPM1D* (3 cases). PPM1D suppresses the activation of several DNA damage response mediators; the mutations identified in *PPM1D* enhanced this activity. One of these cases carried a mutation in *PDGFRA* (in addition to *PPM1D*). One had a mutation in *NF1* (in addition to *PPM1D*). Mean age of these adult patients was 29, and the predominant location was in the medulla (four cases) and midbrain (2 cases). Two of the cases had dual location in the medulla and pons. Notably, while all the *IDH1* mutant cases had a pontine location, only 2/7 of the *H3F3A* mutant cases had a pontine location, and the *H3F3A* mutant cases tended to be younger on average. Histopathology in this group was oligoastrocytoma (two cases), astrocytoma (two cases), oligodendroglioma (one case), and glioblastoma (two cases). All *H3F3A* mutations identified in this study involved the K27M amino acid substitution. No *H3F3A* G34 mutation or *HIST1H3B* mutations were detected. *H3F3A* mutations at G34 are typically found in the cerebral hemispheres and occur in adolescence and in young adults, in contrast with *H3F3A* mutations at K27, which are most common in brainstem gliomas of young children, but may also occur in adults as shown by this study (9, 29).

The frequency of *MGMT* promoter methylation in adult brainstem glioma has not been adequately evaluated, although positive MGMT expression (correlating with an unmethylated *MGMT* promoter) was found in 64.7% of cases in one series (31). 1p/19q codeletion is rarely detected in adult brainstem glioma. A genomic profiling study of nine adult brainstem gliomas identified one *BRAF V600E* mutation and two *PIK3CA* mutations (5). Mutations in *AVCR1* (encoding a type I activin A receptor kinase) are commonly seen in pediatric DIPG and may lead to overactivation of the BMP–TGF-β signaling pathway (32, 33). However, the frequency of *AVCR1* mutations in adult brainstem glioma is unknown. Amplification of *MYCN* was found to define a subset of pediatric DIPG in one genomic analysis; the frequency of this finding in adult brainstem glioma is also unknown (34).

## Future Directions

Brainstem gliomas, particularly in adults, are not one disease. Armed with genomic sequencing technology that is becoming increasingly sophisticated and widely available, the future of treatment for adult brainstem glioma lies in parsing out which tumors are most likely to respond to which therapies. Based on current knowledge, for example, it appears that most adult brainstem gliomas can be stratified into one of two groups based upon whether the tumor has an *IDH* mutation or *H3F3A* mutation. Interestingly, *IDH* mutations lead to DNA hypermethylation throughout the genome, while *H3F3A* mutant tumors are associated with DNA *hypo*methylation (30, 35). So while both of these tumor types have a significant epigenetic basis, opposite strategies will be required to target the DNA methylation status of these two groups. Pharmacologic inhibition of histone H3 demethylation has shown promise in preclinical studies on *H3F3A*-mutant brainstem glioma cell lines, and small molecule H3 demethylase inhibitors are in development (36, 37). The multihistone deacetylase inhibitor panobinostat has also been shown to restore methylation and normalize gene expression in preclinical models of DIPG (38). Other studies suggest that somatic histone H3 alterations may result an increased expression of bromodomain-containing protein 1 and 4 (BRD1 and BRD4) (37). Tumors with these mutations may thus be susceptible to treatment with a bromodomain inhibitor, several of which are in development. The subset of brainstem gliomas with *MYCN* amplification may also be susceptible to bromodomain inhibition (39).

Brainstem gliomas that harbor *IDH* mutations, on the other hand, may respond to hypomethylating agents, such as 5-azacytidine and decitabine, both FDA approved for myelodysplastic syndrome. Though not yet confirmed by clinical trials, at least three preclinical studies have demonstrated a therapeutic effect of DNA hypomethylating agents on *IDH1*-mutant tumor cell lines (40–42). In one of these studies, 5-azacytidine was administered intraperitoneally to nude mice bearing subcutaneous xenografts derived from an *IDH1*-mutant anaplastic astrocytoma specimen. At week 14, tumor regression was observed in the 5-azacytidine-treated mice, in stark contrast with the control mice (41). A recent study outlined a novel mechanism involved in the pathogenesis of *IDH*-mutant gliomas: methylation of insulator protein binding sites within DNA, leading to aberrant contact of the *PDGFRA* promoter with distant enhancer elements, causing overexpression of PDGFRA, a known glioma oncogene (42). *In vitro* treatment of *IDH1*-mutant BT142 gliomaspheres with 5-azacytidine reduced methylation of the insulator protein binding site by ~2.5-fold and downregulated PDGFRA expression 5-fold. Phase I pharmacokinetic/pharmacodynamic trials would be needed to confirm the ability of DNA hypomethylating drugs such as 5-azacytidine and decitabine to reach tumors within the central nervous system at therapeutic concentrations.

A phase I trial of AG-120, an oral inhibitor of mutant IDH1, demonstrated stable disease in 10 of 20 patients with *IDH1*-mutant glioma, with a 6-month clinical benefit response rate of 25%. Further testing is ongoing. Other mutations that have been reported in adult brainstem glioma, such as mutations in *PDGFRA, PIK3CA*, and *PPM1D*, are also potentially targetable with drugs that are either already commercially available or in development.

Incorporating genomic data into a strategy for treatment – an approach called “precision medicine” by some – also faces significant challenges. For this strategy to get off the ground, obtaining tumor tissue for genomic analysis is a prerequisite. While operating on the brainstem requires great expertise and care, the precision with which a neurosurgeon can operate these days, coupled with decreasing tissue requirements for genomic analysis, makes obtaining tissue on a standard basis a much more reasonable option now.

Another challenge in adopting a precision medicine approach is figuring out how to incorporate such a strategy into the traditional clinical trial framework. Adopting such methodology, a clinical trial for pediatric patients with DIPG – still ongoing, but not recruiting – uses the result of EGFR and MGMT promoter methylation testing to determine whether patients receive temozolomide and/or erlotinib in addition to bevacizumab and irradiation (NCT01182350). Unfortunately, the incidence of adult brainstem glioma is likely too low for anything larger than a small-scale trial to be performed. However, adults with brainstem glioma may still be candidates for clinical trials as participants in so-called “basket” trials that stratify patients by molecular alteration regardless of tumor histology. For example, adults with recurrent brainstem glioma harboring a *BRAF V600E* mutation may qualify for the recently initiated NCI-MATCH study, which includes an arm for patients with tumors with *BRAF V600E* mutation to receive a combination of dabrafenib and trametinib. Patients may also seek to have genomic profiling performed on their tumor tissue outside the framework of a clinical trial. In these instances, patients must understand that using such data to dictate management is an as-of-yet unproven treatment strategy, promising though it may be.

Immunotherapy may also one day play a role in the treatment of brainstem glioma in adults. Vaccine trials for adult malignant glioma have been conducted for over a decade, but until recently, these trials usually required patients to undergo gross-total or near-gross total resection, which precluded patients with brainstem glioma from participating. Recently, however, some vaccine trials have begun allowing patients with significant residual disease to participate. Vaccines that are manufactured using autologous tumor lysate often require more tissue than is available for patients with brainstem glioma. However, trials that utilize tumor-associated peptides usually have much less restrictive tissue requirements.

Immune checkpoint inhibitors that block targets such as PD1, PDL1, and CTLA4 have recently shown impressive activity across a range of advanced malignancies. These therapies have not yet been evaluated for brainstem glioma, though interest is high.

## Conclusion

Because adult brainstem glioma is a collection of clinical and histological entities, individual patient treatment depends on symptoms, performance status, age, histology, molecular pathology, and availability of new therapeutic studies. IDH and histone mutations are relatively recent discoveries and are only now being introduced into upcoming pediatric studies; retrospective analyses are underway in selected adult patient populations. Given the rapid advances in the field in molecular classification, epigenetics and cancer therapeutics, it is likely that we will make significant gains in this vein over the next decade, thereby enabling evidence-based management algorithms for both pediatric and adult DIPG as well as the larger group of brainstem tumors. For now, we judiciously biopsy/resect patients whom we deem safe to do so for the purpose of symptomatic control and molecular testing for prognosis as well as for enrolling into therapeutic studies which usually require tissue diagnosis and mutation status (e.g., IDH1/2 mutation, BRAF mutation, Histone 3 mutation). Radiation and temozolomide are used routinely upfront and re-radiation is often offered at recurrence. We still have much to learn about the pathogenesis of adult brainstem glioma, but landmark studies on pediatric DIPG on one side and adult non-brainstem glioma on the other is likely to have a considerable degree of relevance. As our understanding of adult brainstem glioma increases, better treatments are sure to follow.

## Author Contributions

JH, SW, and SK contributed to initial and subsequent drafts of the manuscript.

## Conflict of Interest Statement

The authors declare that the research was conducted in the absence of any commercial or financial relationships that could be construed as a potential conflict of interest.
